# Silanes to Silatranes: Robust Functionalization for Single‐Molecule Force Spectroscopy

**DOI:** 10.1002/cbic.202500853

**Published:** 2026-05-10

**Authors:** Thomas D. Courtney, Christopher B. Hatchell, Xuliana O, David R. Jacobson

**Affiliations:** ^1^ Department of Chemistry Clemson University Clemson South Carolina USA; ^2^ Medical Biophysics Graduate Program Clemson University Clemson South Carolina USA

**Keywords:** aminopropyl‐silatrane, force spectroscopy, single‐molecule biophysics, surface chemistry

## Abstract

Atomic force microscopy‐based single‐molecule force spectroscopy (SMFS) experiments require stable and reproducible functionalization of cantilevers and surfaces. Conventional silane‐based methods suffer from poor stability, drastically hindering their reproducibility and performance. Here, we present an ethanol‐based aminopropyl‐silatrane functionalization protocol that achieves superior performance in a similar process timeframe. In particular, combining this approach with bacterial adhesin coupling, we demonstrated a significant twofold improvement in the fraction of attempted SMFS experiments yielding usable single‐molecule data. The method can be generally applied to silicon‐nitride and glass cantilevers and surfaces, including mechanically modified cantilevers optimized for fast time response, low stiffness, and low drift. Furthermore, we demonstrate a simple and cost‐efficient method to clean these functionalized cantilevers using common solvents and a benchtop UV‐ozone cleaner, allowing for refunctionalization and prolonged cantilever lifespans. Together, this allows for a reliable, reusable, and versatile functionalization protocol for high‐quality SMFS experiments.

## Graphical abstract

3‐(aminopropyl)silatrane (APS) has superior stability for surface functionalization in single‐molecule force spectroscopy experiments compared to conventional silane‐based methods. This paper utilizes bacterial adhesin attachment to demonstrate the advantages of APS‐based surface functionalization in single‐molecule unfolding experiments of a model protein, enabling future studies of other biological systems.

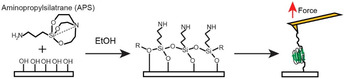

## 1 Introduction

Single‐molecule force spectroscopy (SMFS) is a powerful tool for probing aspects of biological systems including protein folding, ligand interactions, cellular replication, and locomotion. In SMFS experiments, a single biomolecule is tethered between a surface and a force transducer, such as the cantilever of an atomic force microscope (AFM) [1]. Increasing force drives the biomolecule toward unfolded or dissociated states. These states are represented in a resulting force–extension curve (FEC) by characteristic features such as abrupt drops in the force. SMFS can probe systems whether or not they experience force in their biological context [2]. Such studies have reported on conformational dynamics, folding intermediates, and the energetic landscapes of proteins such as titin, α‐helical repeat proteins, and membrane proteins [3, 4, 5, 6, 7, 8]. These studies also can provide insight into genetic diseases that are caused by protein misfolding, leading to potentially improved treatments [9].

To obtain accurate and reproducible data using SMFS, reliable chemical functionalization is needed to site‐specifically attach the single molecule of interest to the surface and the force probe. In such an assay, a protein with an addressable tag is tethered to a surface, and the binding partner of the tag (e.g., biotin‐avidin, cohesin‐dockerin, peptide‐bacterial adhesin [10, 11, 12]) is tethered to the end of the AFM probe (cantilever). Alternatively, site‐specific immobilization can also be achieved via covalent bioorthogonal conjugation strategies (e.g. DBCO‐azide click chemistry), either directly by labeling the sample with a click‐chemistry reagent or by facilitating other enzymatic connections like those mediated by sortase or OaAEP1 [11, 13]. Then, when the probe is lowered into gentle (∼250 pN) contact with the surface, there is some chance of forming a bond between the tag and its partner. This establishes a chemical linkage between the surface and probe, via the protein of interest, that allows for force application to the protein. This site‐specific attachment provides a unique, well‐defined attachment point and reduces artifacts associated with the high surface contact forces of nonspecific adhesion (≳ 1 nN) [1]. Without this site specificity, it can take thousands of attempts to achieve a correct unfolding pattern of a protein [7, 14]. Given that surface and probe functionalization is the first of many steps in performing SMFS experiments with AFM, developing a reliable protocol is crucial.

Over time there have been significant advancements in the reliability and specificity of surface chemistry functionalization. The early approaches usually relied on nonspecific adhesion of proteins to polymer‐coated surfaces [15]. These assays had numerous issues with nonspecific and multiple attachments. Subsequent improvements involved the use of polyethylene glycol (PEG) linkers and specific terminating tags on the protein of interest [1, 10, 16, 17]. Typically, (3‐aminopropyl)triethoxysilane (APTES) or other similar silane derivatives were used to attach biomolecules to glass substrates or to the silicon‐nitride surface of an AFM cantilever. The silane groups of APTES react with free alcohols on a UV‐ozone‐treated surface and form a connected monolayer, which allows for uniform coverage of the surface [18]. The primary amine of APTES then reacts with a functional group on one end of a PEG linker; a maleimide on the other end of the PEG reacts with cysteine residues on the target protein [16]. For a one‐step functionalization, heterobifunctional PEG molecules can be used that already have a triethoxylsilane moiety at one end [19].

**FIGURE 1:**
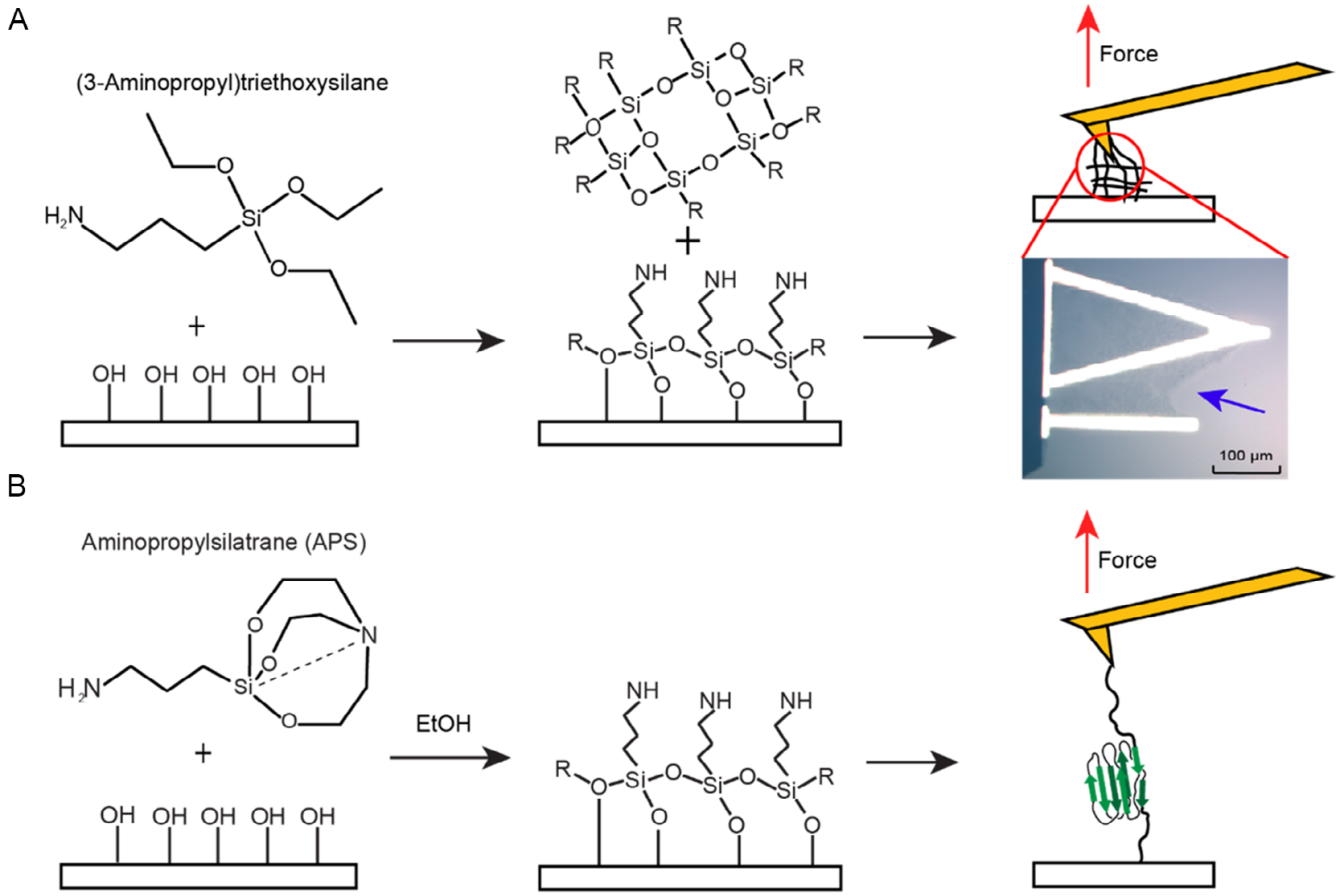
Differing surface‐reaction behaviors of APTES and APS. (A) APTES can react with a UV‐ozone treated glass surface to form the desired surface layer, but can also form crosslinked aggregates that adhere to the cantilever and surface. This can prevent the SMFS study from addressing a single molecule and, in extreme cases, result in films large enough to be seen in visible‐light microscopy of the cantilever (*blue arrow*). (B) APS also reacts with UV‐ozone treated glass to form a surface layer but resists crosslinking in solution. This results in the desired single‐molecule tether.

The practical effectiveness of silane‐based functionalization is, however, compromised by the instability of silane. Due to their structure and sensitivity to water, silanes are prone to hydrolysis and self‐polymerization. The ethoxy functional groups form highly reactive alcohols when exposed to water, which can react with each other (Figure 1A). This can lead to clumping and formation of sticky and uneven layers on a surface [20]. In extreme cases, polymerized silane‐PEG chains can form aggregates on the AFM cantilever on the ∼100‐µm scale (Figure 1A). Also, an uneven monolayer of linker will promote heterogeneous clumping of biomolecules, preventing single attachment to the AFM probe.

3‐aminopropyldimethylethoxysilane (APDMES) has been used with success to address some of the shortcomings of APTES [21, 22, 23, 24, 25]. However, due to containing an ethoxysilane functional group, APDMES can be prone to hydrolysis in aqueous environments, as evidenced by degradation of functionalized surface layers over time [20, 26]. This necessitates specialized storage conditions, such as storage in the absence of solvent under nitrogen atmosphere [26].

To overcome these issues, a compound is needed that has similar reactivity to APTES, in that it can form a stable uniform monolayer, but that will not self‐polymerize in aqueous environments. A strong but underutilized candidate has been 3‐(aminopropyl) silatrane (APS). Although not commercially available, APS synthesis is straightforward and achievable by workers with undergraduate‐level organic chemistry training. APS is structurally similar to APTES but instead of having three loose ethoxysilanes that can be easily hydrolyzed, the ethoxysilanes form a stable tricyclic structure (Figure 1B). The compound will still react with the hydroxyl groups on glass/mica/silicon‐nitride surfaces, but the structure has an intramolecular coordinate bond between the nitrogen and silicon that stabilizes the structure and resists self‐polymerization [27, 28]. To date, APS and other silatrane derivatives have been used to tether biomolecules for AFM imaging and to passivate surfaces [18, 20, 29, 30, 31, 32, 33, 34]. This has included some silatrane‐based SMFS studies done by Lyubchenko and coworkers on peptides using aqueous deposition.

Recent work has shown, however, a critical shortcoming of depositing APS under aqueous conditions: the high solubility of APS in water limits its proclivity to attach to a surface, leading to uneven deposition and incomplete monolayer formation [20]. Andersson et al. showed that they could not achieve the APS layer thickness expected for a completely coated surface using aqueous deposition. However, they showed that a straightforward protocol involving ethanol deposition and a short curing step achieved complete monolayers [20]. While this work was focused on surface passivation, methods enabling complete monolayer formation would also be invaluable to SMFS experiments. Such a uniform layer would help prevent biomolecule congregation caused by uneven surface coverage and allow for the surface density of tethered biomolecules to be solely governed by the concentration of the biomolecule.

This ethanol‐based approach motivated a strategy for improved biomolecule attachment in SMFS experiments. We have previously relied upon silane‐based functionalization following the protocol of Walder et al. [19], which established a simple functionalization procedure using heterobifunctional PEGs with a triethoxysilane moiety at one end and another functional group at the other. However, in our hands, this method resulted in inconsistent functionalization success due to the instability of silanes. Here, by implementing ethanol‐based APS deposition in AFM‐based SMFS experiments (Figure 1B), we achieved remarkably improved functionalization success rates compared to the previous chemistry. In this work we detail the implementation of this method and highlight its advantages compared to previously used silane‐based functionalization, including a dramatically higher rate of successful functionalization combined with consistent, single‐molecule attachment efficiency. We further characterize the chemistry's surface properties on our glass surfaces using contact‐angle goniometry. We also discuss how the method can be extended to functionalize cantilevers that have been previously physically modified to optimize their mechanical properties and to enable reuse of cantilevers by removing the functionalization.

## 2 Methods

### 2.1 Expression and Purification of SdrG and ddFLN4

All proteins were engineered with a C‐terminal cysteine for thiol‐maleimide coupling and an N‐terminal 10x histidine tag for purification. Plasmids encoding staphylococcal adhesin protein SdrG and Fgβ‐tagged *Dictyostelium discoideum* domain 4 (ddFLN4) were used to transform *E. coli* BL21(DE3) cells, which were then induced to express the respective proteins. Following Milles et al. [10], cultures were grown in 1 L of kanamycin‐containing LB broth for 16 h at 37°C under 250‐rpm shaking. 1 mL of 1 M IPTG was added, and the solution was shaken for another 3.5 h until growth became stationary. The culture was centrifuged for 10 min at 8,000 g. The resulting pellet was suspended in 30 mL of lysis buffer (50 mM 7.4 pH tris, 50 mM NaCl, 5 mM MgCl_2_, 0.1% Tween‐20, 10% glycerol, and 100 µg/mL lysozyme). Cells were lysed through sonication at 40% duty cycle, 8 s on and 8 s off for 120 s (Fisherbrand Model 505). The lysed cells were centrifuged again at 20,000 g for 45 min. The resulting supernatant was collected and purified in a metal affinity Ni‐NTA column containing 4 mL of resin. The column was washed with 4 column volumes (CV) of binding buffer (25 mM tris, 500 mM NaCl, 20 mM imidazole, 0.25% Tween‐20, 10% glycerol) before the sample was loaded. The column was then washed with 4 CV of wash buffer (25 mM tris, 500 mM NaCl, 20 mM imidazole, 0.25% Tween‐20, 10% glycerol) and the protein was subsequently eluted with 4 CV of elution buffer (25 mM tris, 500 mM NaCl, 250 mM imidazole, 0.25% Tween‐20, and 10% glycerol). Protein identity was confirmed via SDS‐PAGE. The sample was desalted by centrifugal filtration, and the concentration was determined via a fluorescence assay (Qubit Protein Broad Range Assay, Thermo Fisher Scientific Inc.).

### 2.2 Aminopropyl Silatrane Synthesis

APS was synthesized following Andersson et al. [20]. A one‐pot reaction of APTES (2.2 mL, 10.5 mmol), triethanolamine (0.8 mL, 6 mmol), methanol (7 mL), and NaOH (15 mg, 1.5 mmol) was refluxed for 24 h at 85°C in a round‐bottom flask. After removing the solvent via rotary evaporator, the product was poured into a solution of n‐hexanes (50 mL). The resulting solid was collected by vacuum filtration. After overnight vacuum filtration, 1.013 g of white solid product was obtained (72% yield). The product was dissolved in 3 mL of water to make a 1.435 M solution and stored at 4°C. Identity was confirmed via NMR (Figure S1).

### 2.3 Surface and Cantilever Cleaning and Functionalization

Glass coverslips were cleaned by sonication in acetone for 5 min, followed by sonication in 95% ethanol for 5 min, and then submersion in 3 M KOH solution for 3 min. The slips were then rinsed in water and sonicated in water for 3 min before being dried under a N_2_ stream.

Step‐by‐step methods for surface and cantilever cleaning and functionalization are given in the Supporting Information. Briefly, surface functionalization began with UV‐ozone cleaning (PSD Pro Series Digital UV Ozone System, PSDP‐UVD8, Novascan Technologies Inc.) for 30 min. Following treatment, a droplet of APS from a 91 mM APS solution in 99% ethanol was placed on each surface for 60 s. The surface was then rinsed in ethanol and dried with a gentle N_2_ stream. Surfaces were then placed on a preheated 75°C petri dish to cure for 60 s. Following curing, the surfaces were rinsed in water, dried under N_2_, and placed in a 0.15 mg/mL solution of NHS‐PEG‐maleimide (1 kDa MW, Nanosoft Polymers) for 1–2 h. After deposition, the surfaces were rinsed in toluene, isopropanol, and water (20–30 dunks for each solution) and dried before adding a 50 µL droplet of protein in tris‐NaCl buffer (50 mM tris, 150 mM NaCl, pH 7.4) and storing at 4°C in a humidity box until use.

Prior to functionalization, cantilevers were first rinsed for 30 s each in toluene, isopropanol, and water. They were then treated with UV ozone for 30 min. Following treatment, each cantilever was placed into a droplet of 91 mM APS solution in 99% ethanol for 45 s. They were then rinsed in ethanol and dabbed dry with a lint‐free wipe and placed on a preheated 75°C petri dish to cure for 45 s. After curing, the cantilevers were rinsed in water, blotted dry again, and placed into a solution of 0.15 mg/mL NHS‐PEG‐maleimide in toluene with 2 mL of isopropanol at 60°C for 1–2 hrs. The cantilevers were then rinsed in toluene, isopropanol, water, and then isopropanol for 30 s in each solution and then blotted dry with a lint‐free wipe before being placed in a 50 µL droplet of protein in tris‐NaCl buffer (again 50 mM tris, 150 mM NaCl, pH 7.4) and stored at 4°C in a humidity box until use. When functionalizing soft, mechanically modified cantilevers, additional caution was needed to prevent cantilever bending due to capillary forces, including drying under N_2_ rather than by dabbing. These modified procedures are discussed below and in Supporting Method 1.

### 2.4 SMFS Assay

**FIGURE 2:**
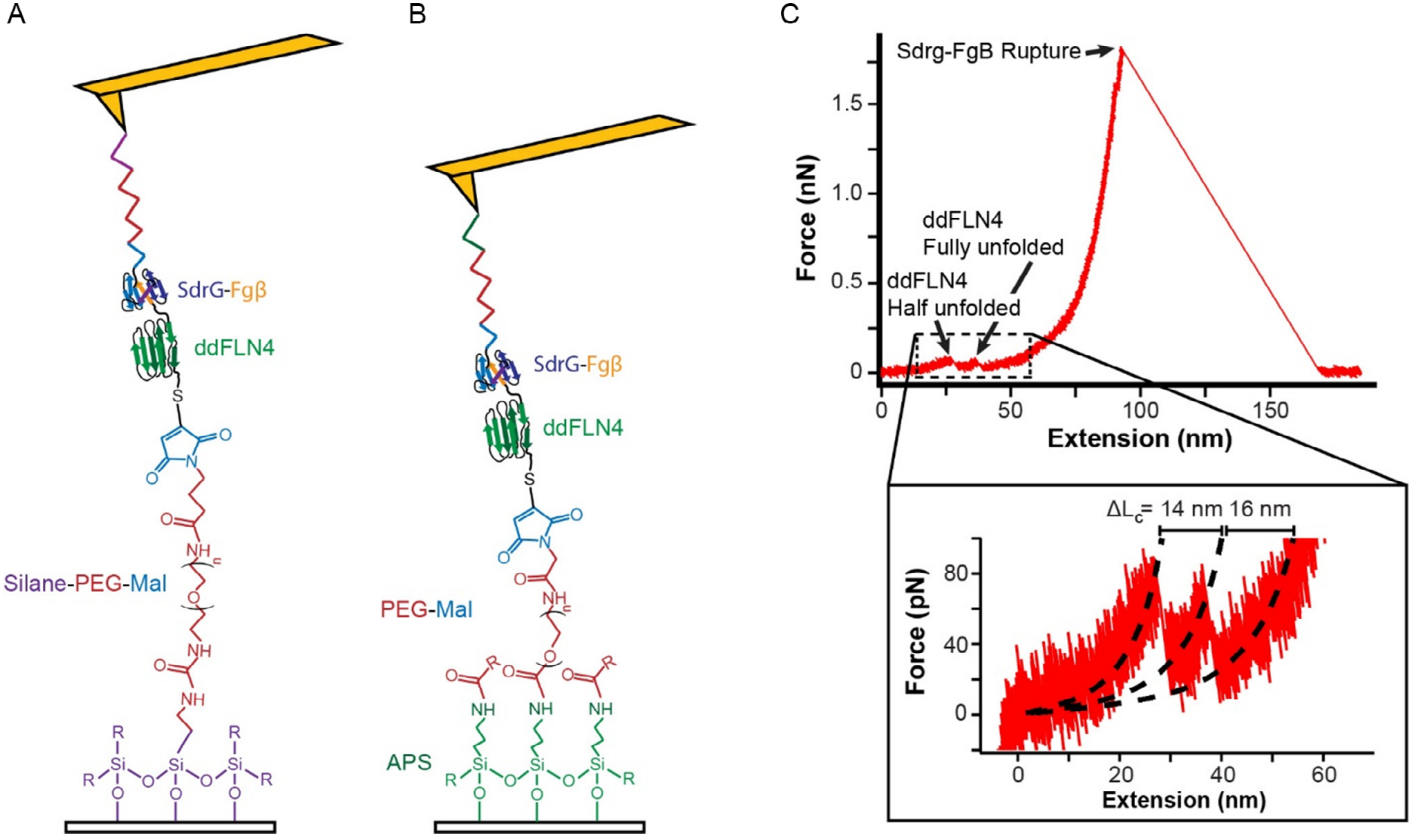
SMFS protein‐unfolding assay of ddFLN4 implemented using either (A) SPM or (B) APS chemistry. In each, it is the noncovalent SdrG‐Fgβ linkage that forms during a single‐molecule pulling event. Detailed chemical structures for attachment of ddFLN4 to the substrate are shown; identical chemistry is used to connect SdrG to the AFM probe (*colored squiggle*). Not drawn to scale and drawn with simplified protein representations. (C) FEC of ddFLN4 is shown with labeled unfolding intermediates as well as SdrG‐Fgβ rupture. (*Inset*) Fit of ddFLN4 folded, intermediate, and unfolded states using WLC model confirms expected contour‐length changes ($\text{Δ}L_{c}$). Data sampled at 50 kHz.

Force‐extension data were taken using a Cypher ES AFM (Asylum Research). The cantilevers used were Bruker MLCT‐BIO (tip B, nominal spring constant: 0.020 N/m). Force‐extension data were taken at 300 nm/s velocity with 250 pN surface contact force. Surfaces were probed in randomly selected 20 × 20 µm regions. In each region a FEC was acquired at evenly spaced positions in a grid of 400 points. After 400 points, a new region was probed elsewhere on the surface. We considered a functionalization successful if it yielded at least one record with the correct unfolding pattern of a single molecule of ddFLN4 and the subsequent rupture of the SdrG‐Fgβ interaction (Figure 2C). To check for the correct unfolding pattern, we looked for two unfolding transitions that are indicative of ddFLN4's unfolding pathway and fit them with a worm‐like chain (WLC) model. In this model the elasticity was treated as the sum of the unfolded amino‐acid chain elasticity modeled using the Marko–Siggia WLC interpolation formula with 0.4 nm persistence length and the additional elasticity of the PEG linker modeled using the freely jointed chain model of Oesterhelt et al., which accounts for PEG structural isomerization [35, 36, 37].

### 2.5 Cantilever Refunctionalization

Previously functionalized cantilevers were first rinsed for 30 s in water, followed by rinsing for an additional 30 s each in toluene, isopropanol, and water. They were then treated with UV ozone for 30 min, subjected to another 30 s rinse in each of toluene, isopropanol, and water, and then treated for a further 30 min under the UV‐ozone cleaner. At this point the cantilevers were treated with the normal functionalization procedure described above, starting with APS droplet deposition.

### 2.6 Contact Angle Measurements

Static water contact angle measurements were performed using a DSA 20E goniometer (Krüss) at 20°C. Measurements were made via the sessile drop method [38]. Approximately 1.5 µL was dispensed onto each surface using an automated dosing system. Images of the droplet's profile were taken immediately after deposition. Contact angles were measured using instrument software by either baseline fitting or ellipse fitting method depending on droplet shape. The software recorded three values: left contact angle, right contact angle, and the mean contact angle.

### 2.7 Statistical Analysis

All force‐distance curves were analyzed using Igor Pro 9. Baseline correction was performed by calculating the average force in a noncontact region of the curve and subtracting that value from all other points to set our zero‐force level. To convert force‐distance curves to force‐extension curves, cantilever deflection was subtracted from the raw displacement value.

**FIGURE 3:**
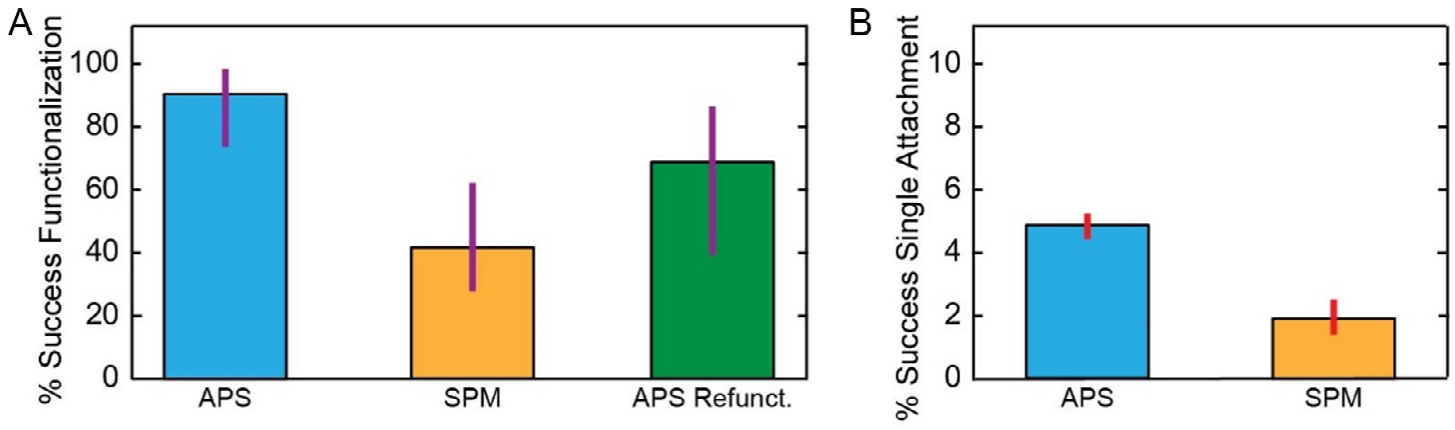
(A) Success rates across functionalization attempts for APS chemistry, APTES/SPM chemistry, and refunctionalization with APS chemistry. 95% confidence intervals shown as purple lines. (B) Attachment success rates across single‐molecule pulling attempts for both chemistries. Red bars denote standard errors of the mean across multiple trials.

All force‐distance curves collected were analyzed and considered in our data in Figure 3A. 95% CI for success rates were determined using the Wilson score method. 28 independent experiments, each collected from a new functionalized cantilever, were analyzed for the SPM chemistry results for a 42% success rate (95% CI: 27‐61%; *n* = 28 AFM probes) and 24 independent experiments for the APS chemistry for an 88% success rate (95% CI: 69‐96%; *n* = 24). For refunctionalized cantilevers, 10 cantilevers previously functionalized with APS were refunctionalized (*n* = 10). Successful curves for all three datasets were identified using the criteria described in the SMFS Assay section, above. For detailed analysis in Figure 3B, data were selected from the four independent SPM experiments and six independent APS experiments that yielded the highest number of analyzable force‐distance traces, *n* = 3,263 and *n* = 9,130 respectively. The resulting single‐molecule attachment rates are reported as the mean of the per‐probe success rates, with uncertainty quantified as the standard error of the mean.

Table 1 used the same success criteria as above. Three tips were used (*n* = 3) on surfaces treated with differing protein concentrations: 1.16, 11.6, and 116 µM. From these, 807, 849, and 802 traces were collected, respectively.

**Table 1:** Effect of protein concentration on single‐molecule attachment rate.

Concentration (µM):	Total attempts	Single attachment %	Multiple attachment %	Ratio of single to total attachment (%)
1.16	807	1.9	1.1	62.5
11.6	849	2.6	1.8	60.0
116	802	0.5	7.6	6.2

Contact angle measurements were taken once on each surface for a total of *n* = 3 surfaces. The results reported are mean contact angles, with uncertainty quantified by the standard deviation of the distribution.

## 3 Results and Discussion

### 3.1 Ethanol‐Based Silatrane Deposition Increased Functionalization Success

Silane‐based functionalization strategies, while sometimes effective, can be compromised by the instability of silane. The susceptibility to crosslinking can cause there to be sticky layers of polymer that will increase adhesion to surfaces, rendering most force extension curves uninterpretable. We found that this crosslinking can become so severe that a visible layer of polymer can form on the cantilever (Figure 1A). This led us to implement a new functionalization strategy based on Andersson et al. [20], utilizing silatranes deposited in ethanol rather than silanes. We investigated the success of this new approach by comparing it to an existing functionalization strategy in protein SMFS measurements.

Our prior functionalization strategy involved binding triethoxysilane‐PEG‐maleimide (SPM) to a UV‐ozone cleaned‐glass surface or silicon‐nitride AFM probe (Figure 2A) [19]. The triethoxysilane forms silanols, which readily react with other silanols on the glass surface (or other silanols in solution); the maleimide undergoes a Michael addition to react with a thiol group on the terminal cysteine of the target‐tagged biomolecule. SPM was similarly used to attach the cysteine tagged binding partner to the AFM probe. We utilized the bacterial adhesin SdrG and its target tag, the 15‐amino acid Fgβ (NEEGFFSARGHRPLD) to tether ddFLN4 to the force probe during a pulling attempt. SdrG binds to Fgβ via a ‘dock lock and latch’ mechanism, forming a remarkably strong catch bond that can withstand forces up to 2 nN [10]. This rupture force exceeds the ∼45 pN unfolding force of ddFLN4. The reversibility of the catch bond at low force means thousands of unfolding events can be attempted per functionalized cantilever. The SdrG‐Fgβ rupture is easily distinguished in the unfolding FEC (Figure 2C), giving further evidence of correct attachment. While we used the SdrG‐Fgβ system in this work, other adhesin‐tag pairs can be used, such as the ClfB‐DK system for pulling from the C‐terminus of a protein [10].

Our new functionalization strategy utilizes the same tags but, rather than SPM, deposits APS onto the surface or probe in an ethanol solution (Figure 2B). Due to its cage‐like structure, APS contains a strong intramolecular donor–acceptor interaction between its nitrogen and silicon atoms, which causes it to be less prone to hydrolysis compared to triethoxysilane [31, 32]. The lone pair on the nitrogen also increases the proton affinity of the oxygens to which it is bound, allowing for increased attraction to the silanol surface compared to other silane derivatives [39]. A proposed mechanism for the reaction suggests that the silicon‐bonded oxygens deprotonate the surface alcohols. The resulting negatively charged surface oxide then attacks the silicon, causing a cascade of similar reactions until the silicon has bonded to three adjacent surface oxygens and a triethanolamine is eliminated [31]. A more detailed reaction scheme is shown in Scheme S1. The primary amine attached to the surface can then readily react with the NHS‐ester end of an NHS‐PEG‐maleimide molecule, resulting in NHS being removed and a bond formed between the amine and the carbonyl of the ester. With the PEG connected to the surface, the maleimide at its other end can then react in the same way as it does in the SPM chemistry (above) to attach to the terminal cysteine of the biomolecule of interest. It is also possible to generalize this approach, using NHS‐PEG molecules with other functional groups besides maleimide, such as click‐chemistry reagents.

We applied both chemistries to an SMFS unfolding experiment of the protein domain ddFLN4, which has a well‐studied and easily identified two‐step unfolding pattern (Figure 2C) [10, 37]. Either the SPM‐ or APS‐based functionalization strategy was used to attach SdrG to the AFM probe and Fgβ‐labeled ddFLN4 to the glass substrate. For the SPM chemistry, we observed a success rate of 42% (95% CI: 27‐61%; *n* = 28 AFM probes), while the APS chemistry had a significantly higher success rate of 88% (95% CI: 69‐96%; *n* = 24) using the Wilson score confidence intervals (CI) (Figure 3A) [40].

We also tested the ability of each functionalization to yield numerous single‐molecule attachments in long‐duration experiments when protein was deposited on the surface at ∼10 µM concentration. We found that the SPM chemistry yielded a mean single‐molecule attachment rate of 1.9% ± 0.8% and the APS chemistry yielded 4.8% ± 0.4% (mean of per‐probe success rates ± standard error; *n* = 4 probes for SPM and *n* = 6 probes for APS). These results were calculated from a total of 3,263 (SPM) and 9,130 (APS) pulling attempts. In this process we did see that there was a drastic increase in multiple attachments in the APS chemistry. One exemplary APS‐functionalized cantilever showed 224 total attachments out of 1712 attempts, with 92 of those being single attachments and 132 being attachments to more than one molecule at the same time. This shows there is further room for improved single‐molecule (vs. multiple‐molecule) attachment rate by tuning the surface concentration.

To evaluate the relationship between deposited protein concentration and the frequency of single attachment, we performed an experiment with three tips from the same functionalization batch at three different protein deposition concentrations: 1.16, 11.6, and 116 µM. With each tip, we made approximately 800 pulling attempts. As summarized in Table 1, both single and multiple attachment events increased with increasing concentration; however, the proportion of single‐molecule events changed distinctly above a threshold concentration. At 1.16 µM and 11.6 µM, about 60% of total attachment events were with one molecule at a time. In contrast, at 116 µM only 6.2% of interactions were with single molecules. The significant majority of interactions at that concentration were uninterpretable linkages to multiple proteins at the same time. This indicates the presence of a threshold concentration up to which total attachment events can be increased while maintaining an optimal proportion of single‐molecule interactions. Given that ethanol‐based APS deposition produces a uniform surface coating, controlling the surface density of the deposited protein is likely critical for minimizing multiple attachments and improving the single‐molecule yield.

### 3.2 Functionalization of Modified Cantilevers

The quality of SMFS data has been shown to be critically dependent on the mechanical properties of the AFM cantilever [41, 42, 43]. Using focused ion beam (FIB) milling and chemical etching, small, low‐drag cantilevers can be physically modified to reduce their spring constant, resulting in lower force noise and force drift while maintaining fast response times. This allows for high‐precision measurements that can capture previously hidden protein intermediates [44]. Because of their low spring constant, we expected these modified cantilevers to be especially vulnerable to damage during functionalization, particularly when passing through air–liquid interfaces. And, indeed, we found that the functionalization procedure reported in Methods needed to be changed in several ways to prevent irreversible damage to soft, modified cantilevers. In particular, we found it to be essential to dry cantilevers with a gentle nitrogen stream rather than by blotting with a lint‐free wipe. We found the capillary hydrodynamic force during blotting would commonly cause the cantilever to irreversibly bend. We also found the step in which the cantilever is dried prior to curing on the 75°C petri dish to be essential. Supporting Method 1 shows how these steps are integrated into the functionalization workflow. In contrast, none of the unmodified cantilevers exhibited bending behavior during the drying or curing step. Without these steps, the modified cantilever became irreversibly bent away from the horizontal orientation needed to properly reflect the AFM detection laser. With these steps, however, we were able to successfully functionalize and refunctionalize multiple Olympus AC40‐TS cantilevers that were modified according to a previously reported procedure [45]. We observed no noticeable degradation in the modified cantilevers’ crucial mechanical properties of spring constant, resonant frequency, and quality factor across two cycles of functionalization and cleaning (Figure S2).

### 3.3 Cantilever Cleaning and Refunctionalization

Functionalized cantilevers only maintain optimal performance for a few weeks to a month without use and with use can only achieve a finite number of pulls [19]. Due to this, the cost of cantilevers and especially the time investment in FIB‐modifying cantilevers, a reliable method of cleaning functionalized cantilevers for subsequent refunctionalization is desired. A reactive ion etcher has been used to remove PEG coatings from cantilevers in the past; however, such instruments are costly and often housed in specialized facilities like clean rooms [46]. Research by Vig has shown that a variety of surfaces (glass, mica, quartz, and metals) can be cleaned of organic material using a short rinse followed by exposure to UV ozone [47]. The idea behind UV‐ozone cleaning is that organic contaminants absorb in the UV and become excited, dissociating into ions and free radicals. Atomic oxygen and ozone are also formed when O_2_ is exposed to the UV; these then react with the excited organic molecules and ions to form volatile species such as CO_2_, N_2_, and H_2_O [47].

We demonstrated that this UV‐ozone cleaning method, with modifications including introduction of intermediate rinsing steps, effectively removes intentionally deposited organic layers and allows for successful refunctionalization of AFM cantilevers [47]. First, to remove any residual salts, the already functionalized cantilevers were rinsed with water followed by sequential rinsing in toluene, isopropanol, and water. We then treated them in the UV‐ozone cleaner for 30 min. Refunctionalization, however, was not consistent under these conditions. Since prior studies of UV‐ozone cleaning surfaces have not considered a dense silatrane layer, we hypothesized that some of the organic residues may only be partially oxidized or vaporized during the initial UV‐ozone step. We thus introduced a second rinsing step in the same solvents, followed by another 30 min in the UV‐ozone cleaner.

Our assay to demonstrate successful refunctionalization was the same as the APS success‐rate assay. We again asked what percentage of our refunctionalization attempts were able to yield at least one single‐molecule attachment to ddFLN4 with the expected unfolding FEC. Studying cantilevers that had been functionalized at least one time previously and applying our UV‐ozone treatment before refunctionalization (see Methods), we observed a 70% success rate (95% CI: 40‐89%, *n* = 10) (Figure 3A). This rate is consistent with a high rate of refunctionalization success and indicates that this cleaning process is suitable for extending cantilever lifetimes in SMFS experiments. Of the 7 successfully refunctionalized cantilevers, 86% achieved at least one successful single attachment within the first 20–100 pulling attempts, indicating consistent data quality throughout functionalization cycles.

### 3.4 Contact Angle Measurements

To characterize our chemistry's surface properties, we performed contact‐angle measurements using a DSA 20E goniometer. We performed this test on (1) glass surfaces cleaned (but not functionalized) using the KOH‐based process that precedes the functionalization and (2) functionalized surfaces with APS and PEG deposited. For our KOH‐cleaned surfaces, we observed an average contact angle mean of 10.81° ± 0.08° (mean of mean contact angle ± standard deviation; *n* = 3 surfaces), which indicates strong hydrophilicity. Contact angles of our functionalized surfaces were unable to be measured as they fell well below the approximately 10° limit of detection of the instrument. This indicates a further increase in hydrophilicity beyond that seen for bare glass surfaces. This increase in hydrophilicity following pegylation matches previously reported trends [48, 49, 50, 51]. Notably, our surfaces appear to be even more hydrophilic than those seen in literature. Even though the assays are not identical, the significant change in surface properties is consistent with our surfaces having high PEG coverage.

## 4 Conclusion

Here, we showed that ethanol‐based aminopropyl‐silatrane functionalization has vastly improved performance for SMFS experiments compared to previous methods involving silane compounds. APS deposition is more reliable while also maintaining a total time of functionalization of approximately 4 h. This functionalization solves the problems caused by the instability of silanes in a versatile way that can be applied to, at least, all silicon‐nitride and glass cantilevers and surfaces, including modified cantilevers with mechanical properties optimized for high‐quality SMFS data. It is especially versatile when, as in these demonstration experiments, it is combined with a bacterial‐adhesin coupling strategy yielding high attachment forces, rapid off‐rates in the absence of force, and, when genetically encoded into the target protein, high labeling efficiencies [10, 11]. Due to the comparable price to the SPM chemistry reagents, to APS synthesis being a simple one‐pot reaction achievable with the most standard undergraduate organic chemistry teaching lab equipment, and to the stability of APS in aqueous solution at 4°C for extended periods, this is an accessible protocol that can be easily implemented in any research lab. Potential users should thus not be discouraged by the need to synthesize the APS reagent.

We also established a simple, cost‐effective method for cleaning APS‐functionalized cantilevers that allows for repeated functionalization and prolonged cantilever lifespans. By using only common solvents combined with a benchtop UV‐ozone cleaner, this approach can be easily implemented in most laboratories.

## Supporting Information

Additional supporting information can be found online in the Supporting Information section.

## Supporting information

Supplementary Material
