# Supplementary material for: Silanes to Silatranes: Robust Functionalization for Single‐Molecule Force Spectroscopy
Source: Chembiochem. 2026 May 10;27(9):e202500853. doi: 10.1002/cbic.202500853 (PMC13157887; doi:10.1002/cbic.202500853)
Supplement: Supplementary file 1 — Supplementary Material [file CBIC-27-e202500853-s001.pdf]

Supporting Information

Silanes to Silatranes: Robust Functionalization for Single-Molecule Force Spectroscopy

Thomas D. Courtney,[a] Christopher B. Hatchell,[a] Xuliana O,[a] and David R. Jacobson*[a][b]

[a]Department of Chemistry, Clemson University, Clemson, South Carolina 29634, United States

[b]Medical Biophysics Graduate Program, Clemson University, Clemson, South Carolina 29634, United States

*E-mail: djacob4@clemson.edu

Supporting Figures

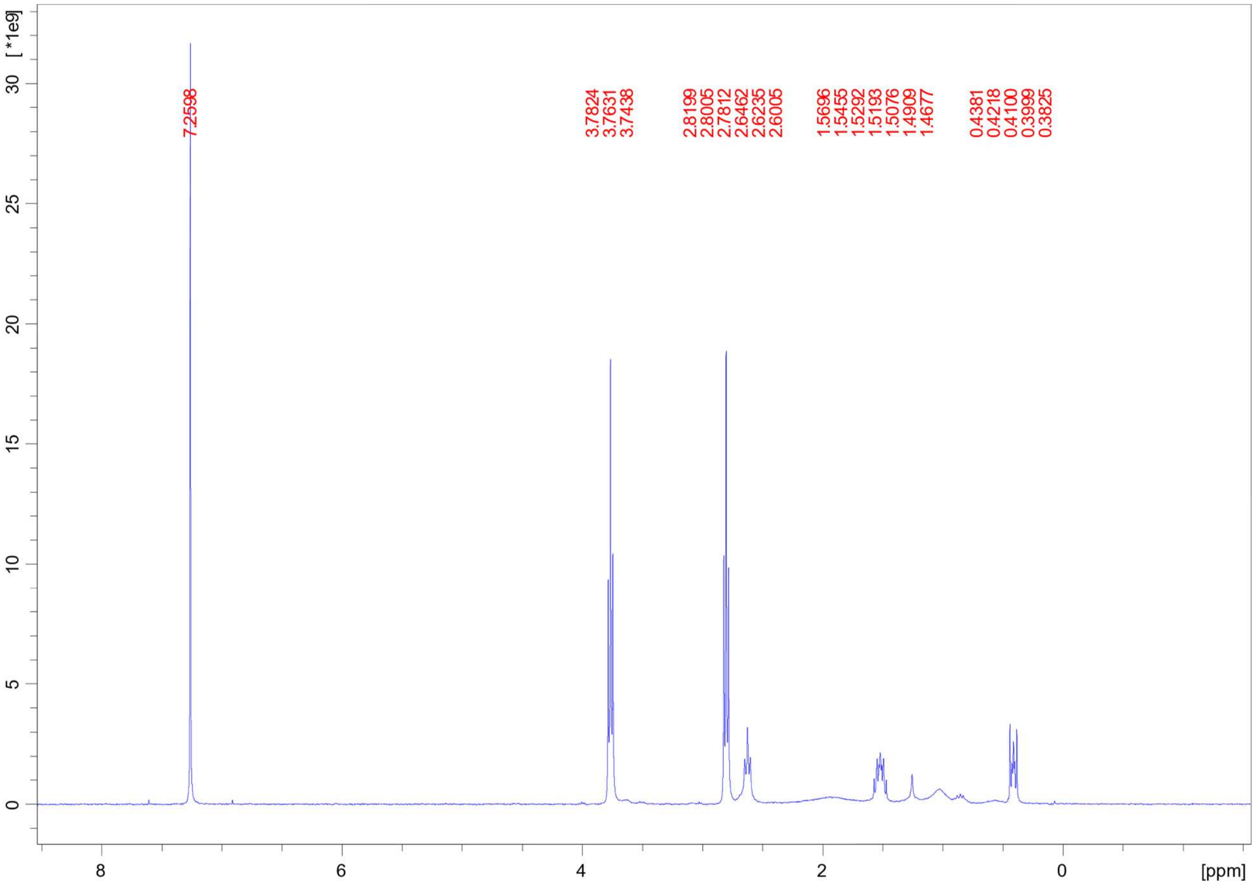

Figure S1: NMR of 3-(aminopropyl) silatrane in $CDCl_3$ (Bruker, 300 MHz)

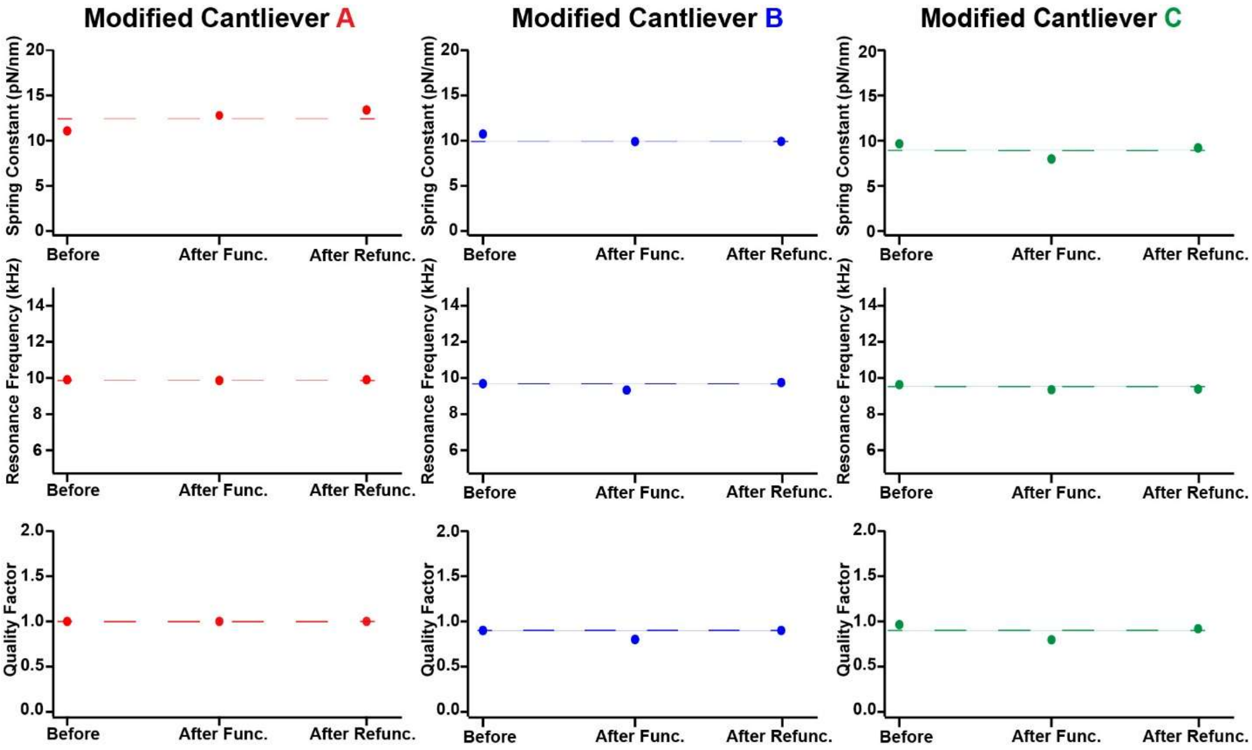

Figure S2: Spring constant, resonance frequency, and quality factor for 3 modified cantilevers before functionalization, after functionalization, and after re-functionalization. These results suggest that functionalization and cleaning do not degrade modified-cantilever mechanical properties.

Supporting Schemes

Supporting Scheme 1: Proposed mechanism of APS reacting with UV-ozone treated silicon nitride surface

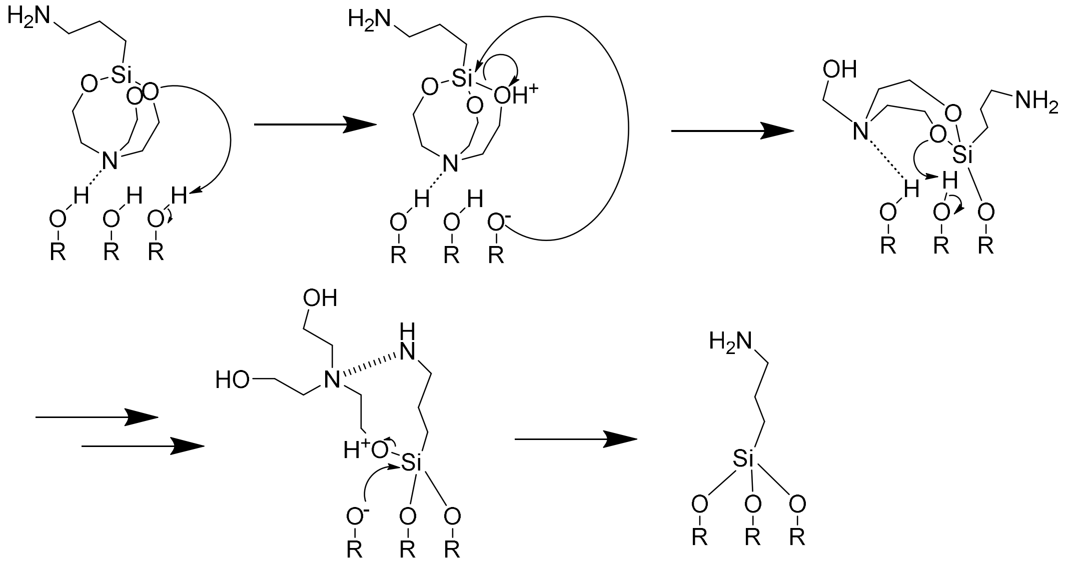

This mechanism was based on proposed mechanism of mercaptopropylsilatrane by W. H. Chen et al.[1]

Supporting Methods

Supporting Method 1: Detailed Cantilever/Surface Functionalization

Important Notes:

- Condition all beakers by pre-rinsing with desired solvents
- All temperatures given are the temperatures of the solution itself, so hot plates may need to be adjusted accordingly
- To follow this procedure, surfaces should have already undergone sonication and KOH cleaning (see Supporting Method 2)
- For soft modified cantilevers, the instructions given in **bold text** should be followed to prevent irreversible cantilever bending.
- **Modified cantilevers should never be dried by blotting with lint-free wipes**

Procedure:

1. Set hot plate to appropriate temperature such that a petri dish placed on it reaches 75°C (Use thermocouple thermometer to check temperature of petri dish).
2. Rinse desired cantilevers for 30 s each in:
    a. Toluene.
    b. Isopropanol.
    c. Ultrapure water (should be used in all steps requiring water and in preparation of all water solutions/buffers).
3. After dipping cantilever once more into isopropanol, dab dry with lint-free laboratory wipe (**Note: Modified cantilevers should be dried via N_2 stream)** and place into clean petri dish. Similarly place any surfaces to be functionalized in a separate petri dish.
4. Load both petri dishes into UV/ozone cleaner and clean for 30 minutes. Note: it may be necessary to adjust cleaning time based on UV light intensity and distance of materials from the lamp.
5. During the UV/ozone cleaning prepare the following:
    a. Set another hot plate such that a glass vessel placed on it reaches 60°C.
    b. Prepare APS solution (combine 20 µL of APS stock 1.435 M with 300 µL of 99.5% ethanol). Will need about 50 µL per each cantilever and surface
    c. Create solution of Mal-PEG-NHS in toluene:
        i. Take out Mal-PEG-NHS from -20°C freezer and let it come to room temperature before opening. Keep it in foil to prevent light exposure. Weigh out approximately 10 mg into a small glass vial or beaker.
        ii. Make sure Mal-PEG-NHS vessel is covered to prevent dust contamination.
        iii. Calculate toluene amount needed to create a 0.15 mg/mL solution of MAL-PEG. Have this volume of toluene standing by in a graduated cylinder.
6. 15 min before UV/ozone cleaning is finished:
    a. Fill a 10 mL beaker with ethanol.

b. Fill a 10 mL beaker with water.
c. Pipette 2 mL of isopropanol into the Mal-PEG-NHS vessel and stir at 400 rpm for 2 min at 60°C on the hot plate.
d. Pipette 2 mL of toluene from the graduated cylinder into the glass vessel and increase stirring to 1000 rpm for a further 5 min (60°C).
e. Pour vessel contents (minus the stir bar) into 250 mL beaker alongside rest of measured toluene. Heat solution to 60°C and stir at 200 rpm.

NOTE: All parts of steps 7 or 8 should be done immediately, one after the other. The timing for this procedure is extremely important to achieve high-coverage, single-monolayer functionalization.

7. For cantilevers:
   a. Once UV/ozone cleaning is finished, deposit a 50 μL droplet of ethanol-APS (prepared above) into a wafer shipper (product 1395-10, Ted Pella Inc.).
   b. Use tweezers to gently deposit each cantilever into a droplet, let sit for *exactly* 45 s. **For modified cantilevers, at 45 s, add 150 μL of ethanol to the wafer shipper before removing it from the APS solution.** (Note: A new droplet should be used for each tip being functionalized)
   c. Immediately rinse tip in 10 mL ethanol beaker and use a lint-free laboratory wipe to gently blot dry the cantilever (touch wipe to back side of chip, away from the cantilever itself). **For modified cantilevers, dry with an N_2 stream.**
   d. Place onto petri dish on 75°C hot plate to cure for *exactly* 45 seconds.
   e. Rinse tip in 10 mL water beaker, gently dry with lint-free wipe, and place into a 60°C petri dish containing the Mal-PEG-NHS/isopropanol/toluene solution prepared above. **For modified cantilevers, after rinsing with water, dip for a few seconds in isopropanol, dry with an N_2 stream and place into the 60°C petri dish containing the Mal-PEG-NHS/isopropanol/toluene solution.**
   f. Let sit for 1–1.5 hrs.
8. For surfaces:
   a. Once UV/ozone cleaning is finished, taking one surface at a time: deposit 50 μL of APS solution onto surface and make sure it covers the whole surface (may need to spread the solution using the tip of a pipette).
   b. Let sit for *exactly* 60 s before rinsing surface in 10 mL ethanol beaker and then gently drying with N_2 stream.
   c. Place onto heated 75°C petri dish to cure for *exactly* 60 s.
   d. Rinse surface in 10 mL water beaker and gently dry with N_2 stream.
   e. Place surface in a polypropylene rack (e.g., Wash N'Dry Cover Slip Rack, Electron Microscopy Sciences) and submerge into 250 mL beaker containing MAL-PEG-NHS/isopropanol/toluene solution that is being heated and stirred at 60°C and 200 rpm.
   f. Incubate with continued stirring for 1–1.5 hrs.
9. 20 min before the end of the 1–1.5 hrs, prepare three 50 mL beakers containing toluene, isopropanol, and water.

10. For cantilevers:
    a. Prepare wafer shipper with a ~50 μL droplet of buffer containing the protein that is to be coupled to the cantilever. When using thiol-maleimide coupling, it is important to attach the protein to the cantilever/surface immediately following functionalization, since maleimide will hydrolyze in the presence of water.
    b. Rinse each cantilever in this order: toluene, isopropanol, water, and isopropanol. Allow cantilever to sit for approximately 30 s in each solvent. Place into the 50 μL droplet.
    c. Leave closed shipper in humidity box at room temperature for at least 1 hr (can be overnight) and then put box into 4°C refrigerator.
11. For surfaces:
    a. Prepare a humidity box with a layer of PTFE tape (smooth side facing up) that the surfaces can be set to rest on after rinsing.
    b. Rinse (by dunking 20–30 times) each surface in this order: toluene, isopropanol, and water. Note: after last water dunk, remove from solvent slowly to try to have as little water as possible remain on the surface.
    c. Immediately put the surface on the Teflon strip in the humidity box and deposit 50 μL of buffer containing desired protein onto surface. Be sure to spread out the solution over the entire surface with end of pipette tip.
    d. Store in humidity box in 4°C refrigerator until use.

Note: for all tips and surfaces, if being left in refrigerator for multiple days, be sure to check every 2–3 days on the droplets and amount of solution on the surfaces and add as needed to make sure everything stays moist.

Supporting Method 2: Glass surface cleaning protocol

Procedure:

1. Condition coverglass holders by squirting with isopropyl alcohol and then drying under a nitrogen stream.
2. Set up bath sonicator (Branson 5200) in fume hood with four 1 L beakers supported with lab clamps. Fill beakers with acetone, 95% ethanol, KOH solution (see below), and water. Also prepare a separate beaker of water.
3. Load circular cover slips (product 26023, Ted Pella Inc.) into holder, then:
    a. Sonicate in acetone for 5 min.
    b. Sonicate in 95% ethanol for 5 min.
    c. Soak in KOH solution for 3 min.
    d. Dip for a few seconds in water (not in sonicator).
    e. Sonicate for 3 min in a separate beaker of water.
    f. Blow slides dry in holder in N_2 stream.

KOH Solution (3 M):

1. Put large stir bar into 1 L washed beaker.
2. Weigh out 50 g KOH pellets.
3. Add 170 mL 95% ethanol, 80 mL water.
4. Cover with aluminum foil.
5. Stir on hot plate (no heat) until ready for use.

Supporting References

[1] W.-H. Chen, Y.-T. Tseng, S. Hsieh, W.-C. Liu, C.-W. Hsieh, C.-W. Wu, C.-H. Huang, H.-Y. Lin, C.-W. Chen, P.-Y. Lin, L.-K. Chau, *RSC Adv.* **2014**, *4*, 46527-46535.
